# Development of a Novel Contactless Mechanocardiograph Device

**DOI:** 10.1155/2008/436870

**Published:** 2008-05-15

**Authors:** Kouhyar Tavakolian, Faranak M. Zadeh, Yindar Chuo, Ali Vaseghi, Bozena Kaminska

**Affiliations:** Computational and Integrative Bio-Engineering Research laboratory (CIBER), School of Engineering Science, Simon Fraser University, 8888 University Drive, Burnaby, BC, Canada V5A 1S6

## Abstract

A novel method of detecting mechanical movement of the heart, Mechanocardiography (MCG), with no connection to the subject's body is presented. This measurement is based on radar technology and it has been proven through this research work that the acquired signal is highly correlated to the phonocardiograph signal and acceleration-based ballistocardiograph signal (BCG) recorded directly from the sternum. The heart rate and respiration rate have been extracted from the acquired signal as two possible physiological monitoring applications of the radar-based MCG device.

## 1. INTRODUCTION

**Figure 1:**
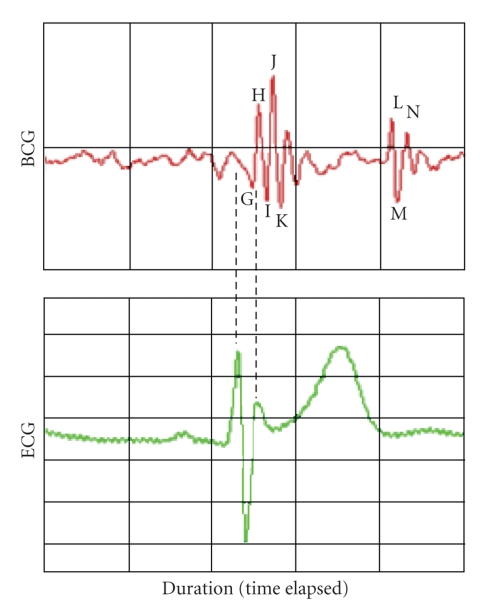
BCG and ECG signals of a single heart-cycle as recorded by the microsensor with the annotation referenced to ECG.

The ballistocardiograph (BCG) is a vital signal in the 1–20 Hz frequency range which is caused by the movements of the heart and blood. The BCG can be recorded from the surface of human body by noninvasive means. In the early 1930s, Isaac Starr recognized that the BCG signals closely reflect the strength of myocardial contraction and classified them into four groups to distinguish normal and abnormal heart performances [1, 2]. As a result of his valuable research, clinicians and medical experts for almost four decades studied the effects of different heart malfunctions by means of the BCG and proved that these malfunctions can be related to typical patterns in BCG signals [3, 4]. The ideal BCG waveform consists of seven waveform peaks labeled H through N as defined by the American Heart Association, and this annotation can be seen in Figure 1.

In addition to a number of clinical studies that have been performed with the BCG, specialized BCG instruments, including beds and chairs, have been developed by different research groups [5–7]. Due to the unrefined nature of the previous BCG signal acquisition technologies, the lack of interpretation algorithms and the lack of practical devices, the current health care systems do not use BCG for clinical purposes. New microsensor and digital wireless technologies provide more accurate BCG signal acquisition and processing, therefore, opening new possibilities to use BCG, and in general mechanical signals of the heart, clinically as a new additional tool in diagnosis and identification of heart malfunctions.

Based on these observations, we are improving the BCG signal acquisition system that reflects the mechanical functioning of the heart. In our first study [8], we used a high-precision accelerometer, which is factory calibrated, weighs 54 g, and is attached to the sternum, the same as used in reference [9]. In our second study, we improved the previous sensor by developing a small mechanically flexible microsensor that could be easily attached to the sternum. It has been proven that all the aspects of a classical BCG signal, (H, I, J, K) waves similar to that measured through a high-precision accelerometer [9], can be identified in the novel microsensor recordings [10].

The current research, reported in this paper, is aiming to go one step further by applying contactless measurement of the mechanical movement of the heart, using a radar-based MCG device operating in the range of 2.45 GHz [11]. The extracted signal is considered a mechanocardiograph (MCG) reflecting the mechanical functioning of the heart and is highly correlated to the BCG signal acquired in previous studies [12].

This paper is organized as follows: in the second section of the paper our signal acquisition methodology is explained and then the comparison of the acquired MCG signal to our previous BCG recordings is presented. In section three, the electronic circuit board design developed for the radar-based MCG device is explained. In section four the results of breathing rate and heart rate measurements using the new technology are presented. The comparison results of the phonocardiograph and the acquired signal are also presented clearly showing the correspondence of our MCG signal to cardiac events.

## 2. MCG SIGNAL ACQUISITION

**Figure 2:**
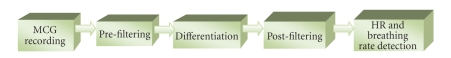
Radar-based MCG signal processing methodology.

The block diagram in Figure 2 shows the general methodology followed in our research for processing of the radar-based MCG signal. The MCG signal was acquired by a microprocessor controlled, radar-based MCG device placed at distance of 10 cm away from the subject's chest. The data acquisition, for the results presented in this paper, involved the measurement of ECG and respiration signals too. These signals were acquired by a Biopac biological data acquisition system [13]. The design principle of radar-based MCG device is presented in section three of the paper.

**Figure 3:**
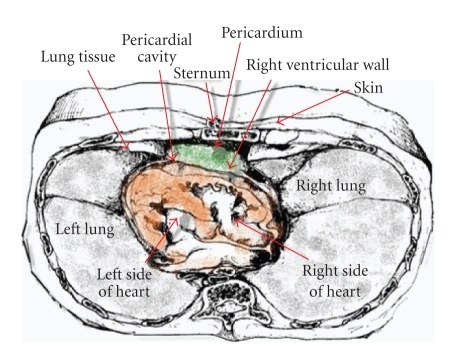
Positioning of different layers of tissues that the radar signal will go through. The layers between sternum and right ventricle include pericardium, pericardial cavity, and thin layers of pleural tissues.

While monitoring the heart from the outside of the body contactlessly from the sternal location, the signal passes through only a few layers of different tissues between the sternum and the heart which can be seen in Figure 3. The tissue layers between the sensor and heart muscle include: skin, sternum, lung and pleural tissue, pericardium and pericardial space. From the sternum position, these tissue layers are thinner compared to the other positions. Therefore, the best position to record the heart's MCG signal is from the sternum (normally on the first third of the sternum length, as this is the closest path to get to the heart muscle).

**Figure 4:**
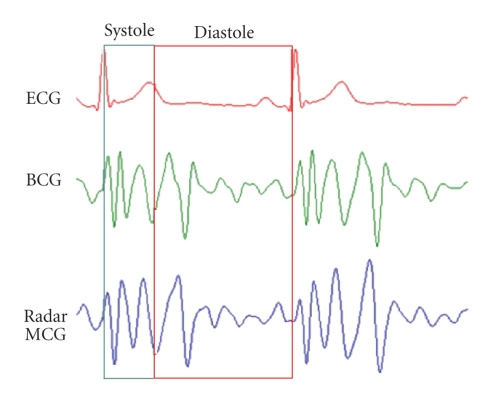
Two cycles of the filtered radar-based MCG signal (bottom), the filtered signal recorded directly from the sternum using a high-precision accelerometer (middle), and the lead I of ECG signal (top).

RF signal with the carrier frequency of 2.45 GHz was transmitted toward the subject's chest, and the reflected signal was band-passed filtered between 0.5 Hz to 25 Hz. The filtered signal was differentiated and then band-pass filtered again between 4 Hz to 20 Hz. The comparison of the processed MCG signal to the BCG signal recorded simultaneously from the sternum can be seen in Figure 4 together with the synchronized ECG signal.

It can be observed that there is a close correlation between the signal acquired from the radar-based MCG device and the signal acquired from the accelerometer-based BCG device attached to the sternum. The systolic and diastolic phases of cardiac cycle are shown to identify the correlations of these mechanical signals to the heart functioning.

Phonocardiograph signal reflects the heart sounds that can be hared using stethoscope. Heart sound S1 corresponds to systolic phase of the heart cycle, and S2 corresponds to the diastolic phase of it. For the comparison purpose, the MCG signal was acquired synchronously with phonocardiograph signal and it was observed that S1 and S2 sounds of the phonocardiograph signal corresponded to the similar complexes on the MCG signal. The clear correlation of this new signal to the heart cycle's events can be observed in Figure 6.

## 3. CONTACTLESS MCG DEVICE

As stated before, in our experiments heart's MCG signal was measured by a MCG device operating in the range of 2.45 GHz. At this electromagnetic frequency, the surface of the body is highly reflective to incident electromagnetic field. Biological tissue is very lossy at this frequency, and there is minimal penetration of radiated electromagnetic energy into the body. Therefore, a return signal from a radiated electromagnetic field incident on the body will primarily contain information associated with movement events.

The effects of radio-frequency fields on human health have been monitored and studied by the scientists around the world for about 60 years now. The 2.45 GHz frequency is considered to be safe for human health. This is the same frequency range as emitted from a cell phone [14]. The specific absorption rate, SAR, is 0.42 w/kg which is below the limits as recommended by international commission on nonionizing radiation protection [15].

**Figure 5:**
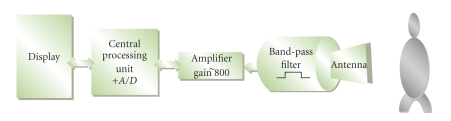
Block diagram of the principle design of the contactless MCG device.

The principal design of the radar-based MCG device is shown in Figure 5. The antenna mounted in the device is HFMD24 by Siemens and contains a transmitter and a receiver in the same housing. The operating frequency is 2.45 GHz. The transmitter transmits continuous wave radio frequency energy towards subject's body. The returned signal is frequency shifted due to the Doppler effect, which is the apparent change in frequency and wavelength of a wave that is perceived by an observer moving relative to the source of the waves. This effect permits the measurement of slight body movements from which physical heart movement signal can be obtained by the MCG receiver.

The output signal from the receiver is filtered and amplified and is sent to the microprocessor for further processing. The cutoff frequencies for the band-pass filter shown in the schematic are 1 Hz and 100 Hz, and the gain of the amplifier is around 800. After filtering and amplifying, the MCG signal is sent to the A/D unit and then to the ATMEL CPU for further processing. The CPU is connected to a thin-film transistor (TFT) display via its serial peripheral interface (SPI) port. The MCG device operates with two AA batteries (2.45 V).

Considering that the MCG device has its own CPU and monitor, it can be used stand alone to acquire and process the MCG signal. There is also another option of sending the data to a personal computer for more advanced processing of the data using Matlab. To have this option on our device, the digitized MCG signal is transformed to packets and sent through UART to the USB and finally to the host personal computer for possible further processing.

## 4. RESULTS AND DISCUSSION

**Figure 6:**
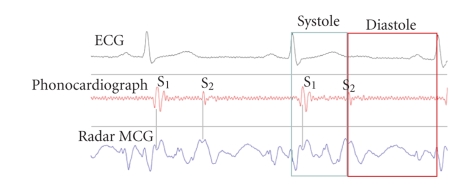
Two cycles of synchronous radar MCG, phonocardiograph, and ECG signal showing the correlation of cardiac cycle events to radar MCG signal. Systolic and diastolic complexes can be identified in the radar MCG signal corresponding to S1 and S2 of heart's sounds.

The data from subjects were acquired at Burnaby General Hospital (British Columbia, Canada) and in all the measurements presented in this paper the distance from the sensor to the chest was set to 10 cm. The processed MCG signal has been superimposed on the phonocardiograph signal, and it has been noticed that S1 and S2 sounds of the phonocardiograph signal corresponded to the similar complexes on the radar MCG signal. The correlation of the MCG signal to the heart cycle's events is observed, and the results of this superimposition can be seen in Figure 6.

For the heart rate measurement our experimental setup included the acquisition of the MCG signal and two leads of ECG as a reference. For respiration rate measurement, the setup included the acquisition of MCG signal together with the respiration signal as the reference. Eight subjects took part in the respiration measurement tests, and six of these subjects took part in the heart rate measurement tests too. Breathing rate measurement experiments were 60 seconds long while heart rate measurement experiments were 15 seconds long.

For detection of breathing rate, the radar signal was low pass filtered under 0.4 Hz, and the peaks were counted and compared to the results acquired from a strain gauge transducer that measures the changes in thoracic circumference, using a belt which is fastened to the subject's thorax. As can be seen in Table 1, the accuracy of respiration rate measurement was 91.35 percent over all eight subjects. The heart rate was measured using radar-based MCG device and was compared to the heart rate calculated from the simultaneous ECG signal for six subjects. The average of the heart rate accuracy on these subjects was calculated to be 92.9 percent.

As an example, for subject six we had 14 cycles of respiration from which 13 of them where correctly identified. Thus, for this subject, the 92 percent accuracy for respiration rate detection was achieved. For the same subject and out of 19 heart beats, 17 of them were correctly identified. Thus, for heart beat detection for this subject, the accuracy of 89.4 percents was achieved. Using our device, the data can be seen real time and can be processed in near real time. The reason for quick processing time is that the algorithm includes filtering of the data and then peak searching to find the corresponding peaks to the inspiration and heart beat events.

The sources of interference for contactless heart motion signal acquisition can be categorized into two main categories. First, foreign electromagnetic radiation that may disrupt the desired signal on its way back to the antenna, and second, motion interference of nearby subjects. As discussed previously, the radiation frequency of the contactless MCG device is centered about 2.4 GHz. This frequency falls within the Industrial Scientific and Medical (ISM) bands. The ISM bands are recognized internationally, where it has been traditionally reserved for industrial, scientific, and medical purposes excluding communications [16].

The 2.4 GHz ISM band in many urban settings is in fact quite saturated with devices. Examples include the very common wireless local area network (WLAN) devices, Bluetooth devices, microwaves ovens, and household cordless phones. These devices can exhibit relatively high signal broadcast power levels during full power transmission. In the North American/US market, the safe power level is governed by the FCC [16]. As a result, it is possible that other 2.4 GHz, ISM devices operating in the vicinity of measurement could pose undesirable electromagnetic signal interference, particularly when these devices are in-motion. However, most cellular phone devices operate in the Global System for Mobile Communications (GSM) Bands, and thus will not pose electromagnetic interference concerns, in the sense of disturbing the Doppler reading, for the contactless MCG device proposed.

 Motion interference is another possible source of signal disruption when operating the device in a high-density, busy setting. The antenna patch on the contactless MCG device is a printed patch antenna and at ranges greater than what is specified operational for this application (i.e., more than 10 cm), its side-lobes begin to pickup motion in its path. Also the main-lobe of the antenna begins to exhibit relatively wide angle. In attempts to acquire useful heart motion signals at longer ranges, more than 10 cm, the motion interference from the subject's general body movement or nearby movements that are not subject-related will both disrupt the heart motion signal acquisition greatly.

In efforts to evaluate the reliability of this device considering the two major sources of interference, in addition to the measurements at Burnaby General Hospital, as explained previously, measurements on 200-plus participants during the Wired NextFest convention were obtained [17]. The environment of the convention hall naturally provided many possible sources of both electromagnetic interference and motion interference. In fact, the convention hosted more than 150 stations, many equipped with audio-video setup, wireless network stations, and up to thousands of visitors at any given moment, likely each carrying some sort of personal mobile device.

The heart motion measurements were compared simultaneously with the corresponding heart beat of each individual participant as assessed via wrist pulse by research staff members. It was observed that when the subject held still, and without other people within 50 cm of the device radius, approximately only one out of ten subjects during 30 second recording session would exhibit grossly delayed or out-of-sync heart rates between the wrist pulse and device reading.

Although it is unknown how many wireless broadcasting devices, using the 2.4 GHz band, were present during the convention, nor the associated power levels, and hence the random interference power density in the foot-ball sized convention hall, the quasi field-test of the device provided assurance that even in the busiest everyday environments, filled with urban electronics and random motion, when the subject recordings are controlled properly, the reliability of the heart rate detection is seemingly good.

Further testing on the effects of both electromagnetic and motion interference is necessary to fully assess the performance of the proposed contactless MCG device. Though, it suffices to say that the observations through the combined evaluations and field-trials give high-hopes on the performance and for the eventual perfection of this device.

## 5. CONCLUSION

In this research work, the MCG signal was extracted from the radar-based MCG device and by using two other heart related signals, BCG and phonocardiograph, it has been proven that the extracted MCG signal corresponds to the mechanical functioning of the heart.

Two physiological signals, heart rate and respiration rate, were measured using the MCG signal and a noticeable accuracy of 91.35% for respiration rate, and 92.9% for heart rate were achieved. It should be noticed that these results are acquired from the first generation of our device, and the accuracy of the detection can be improved by focused targeting of the MCG device on the sternum and further hardware improvements. Another important factor, which is an asset of our device, is the fact that all the electronics and the radar sensor can be fabricated for less than $200 USD which makes our device economical for general heart monitoring purposes.

Considering the short amount of processing and the low cost of the contactless measurement of heart rate and breathing rate as we presented, our device can have numerous applications in the health care system such as, prevention of sudden infant death syndrome (SIDS), sleep apnea, and other areas in which contactless monitoring is desired.

In this research work, we have proposed a new methodology and a new device based on radar technology. We proved the correspondence of our acquired MCG signal to sternal BCG and phonocardiograph signals. Further improvement of the current device can provide us with more accurate MCG signal corresponding to mechanical performance of the heart and ultimately development of an additional contactless heart diagnostic tool.

## Figures and Tables

**Table 1:** The heart rate and respiration rate measurements using the MCG device for eight subjects. The numbers represent the percentage of correctly detected heart beats or breathing cycles to the total number of heart beats or breathing cycles.

Subjects	Heart rate	Respiration rate
1	93	90
2	—	94.2
3	84	90
4	91	90
5	100	87.1
6	89.4	92
7	100	100
8	—	87.5
**Averages**	** 92.9**	** 91.35**
